# miR-128-3p and miR-223-3p as Potential Biomarkers of Metabolic Dysfunction in Liver Tissue from Patients Undergoing Metabolic Bariatric Surgery

**DOI:** 10.1007/s11695-026-08499-3

**Published:** 2026-03-12

**Authors:** Raquel Rodríguez, Daniel Salete-Granado, Lourdes Hernández-Cosido, María Teresa Cabero Morán, Oscar Blanco, Nuria Matesanz, Vanessa Rueda-Cala, Fabiola Campo, María-Ángeles Pérez-Nieto, Carlos Gutiérrez Cerrajero, Edgar Bernardo, Ronald Macías, Guadalupe Sabio, Jorge-Luis Torres, Maura Rojas-Pirela, Miguel Marcos

**Affiliations:** 1 Bariatric Surgery Unit. Department of General & Gastrointestinal Surgery, University Hospital of Salamanca-SACYL, Salamanca, Spain; 2 Department of Surgery, Complejo Asistencial de Ávila-SACYL, Àvila, Spain; 3 University of Salamanca, Salamanca, Spain; 4 Institute of Biomedical Research of Salamanca, Salamanca, Spain; 5 Department of Vascular Biology and Inflammation, Fundación Centro Nacional de Investigaciones Cardiovasculares Carlos III,, Madrid, Spain; 6 Department of Internal Medicine, University Hospital of Salamanca-SACYL, Salamanca, Spain; 7 University of Salamanca, Salamanca, Spain; 8 Foundation Institute for Health Sciences Studies of Castilla y León, Soria, Spain; 9 Organ Crosstalk in Metabolic Diseases Group, Molecular Oncology Program, National Cancer Centre (CNIO), Madrid, Spain; 10 Department of Internal Medicine, Complejo Asistencial de Zamora-SACYL, Zamora, Spain; 11 Pathology Department, University Hospital of Salamanca, Salamanca, Spain

**Keywords:** MiRNA, Obesity, Associated medical problem, Liver, Prognostic and therapeutic tools

## Abstract

**Methods:**

We quantified the expression levels of four miRNAs (hsa-miR-128-3p, hsa-miR-21-3p, hsa-miR-223-3p, and hsa-let-7a) in liver samples obtained during bariatric metabolic surgery from patients with obesity and healthy controls using qPCR. Expression was normalized to hsa-miR-24-3p, selected as the most stable reference gene in our cohort. Associations between miRNA levels and clinical or demographic variables (sex, AMPs) were also explored.

**Results:**

Compared with controls, patients with obesity showed significant dysregulation of hsa-miR-128-3p and hsa-miR-223-3p, independent of histological liver alterations. No consistent changes were observed for hsa-miR-21-3p or hsa-let-7a. Clinical variables, including AMPs, did not account for the observed differences.

**Conclusion:**

miR-128-3p and miR-223-3p are linked to obesity-related metabolic dysfunction and emerge as promising biomarkers or therapeutic targets.

**Graphical Abstract:**

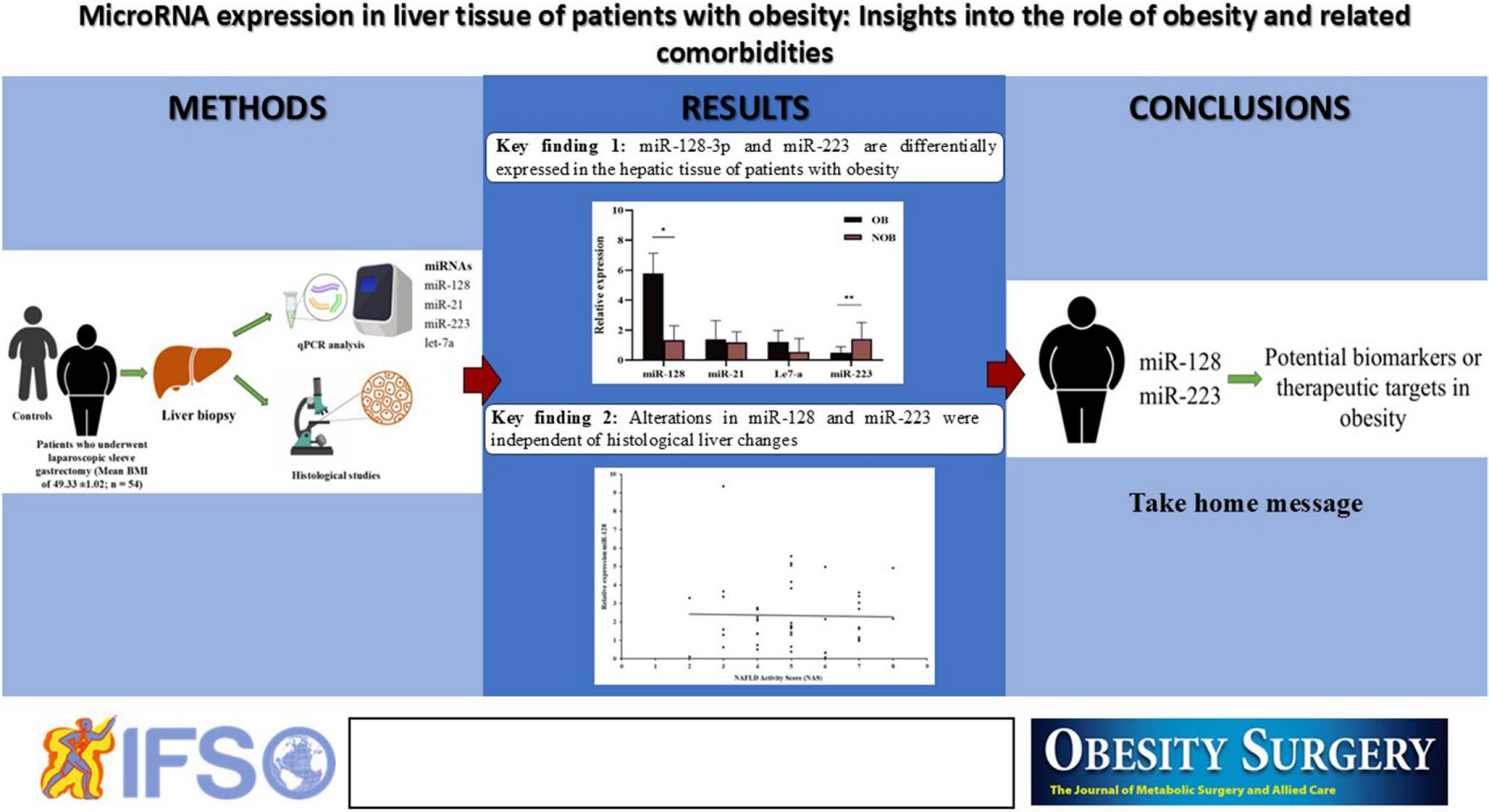

**Supplementary Information:**

The online version contains supplementary material available at 10.1007/s11695-026-08499-3.

## Key Points

Dysregulation of hsa-miR-128-3p and hsa-miR-223-3p was observed in liver tissue from patients with obesity who underwent metabolic bariatric surgery compared to healthy controls.

Alterations of hsa-miR-128-3p and hsa-miR-223-3p were independent of histological liver changes, suggesting a specific link to obesity-related molecular mechanisms.

Hsa-miR-128-3p and hsa-miR-223-3p may serve as potential biomarkers or therapeutic targets for obesity.

**Supplementary Information:**

The online version contains supplementary material available at 10.1007/s11695-026-08499-3.

## Introduction

Overweight and obesity are chronic, relapsing conditions affecting millions of people worldwide. This condition is characterized by abnormal or excessive fat accumulation, which poses a relevant health risk [1]. In 2025, the World Obesity Federation indicates that obesity affects approximately 18% of men and more than 21% of women worldwide, with many countries reporting even higher rates [2]. If current trends continue, projections suggest that by 2050, more than 50% of the global adult population could be affected by obesity [3], highlighting an urgent public health challenge. There are several factors involved in the pathogenesis of obesity in different organs, including alterations in adipose tissue associated with immune cell infiltration, activation of inflammatory pathways, impaired lipid turnover, ectopic fat deposition, and hepatic metabolic alterations, such as dysregulated lipid metabolism, and ultimately, inflammation contributing to metabolic dysfunction [4].

The liver plays a central role in this scenario by undergoing metabolic changes characterized by increased hepatic lipid accumulation (steatosis), insulin resistance, dysregulation of glucose and lipid metabolism, and heightened inflammatory responses. These hepatic alterations not only exacerbate systemic metabolic dysfunction but also contribute to the progression of obesity associated medical problems (AMPs) such as type 2 diabetes and metabolic dysfunction-associated steatotic liver disease (MASLD) [5–7].

In this context, obesity leads to a dysregulation in the expression of microRNAs (miRNAs) in liver tissue, which is closely linked to alterations in the gene expression profiles involved in the development of aforementioned pathogenic changes as well as various other obesity-associated pathologies [8, 9]. miRNAs are a group of small non-coding RNAs that are typically 18–25 nucleotides in length and that post-transcriptionally regulate gene expression. They function primarily by binding to complementary sequences in the 3’-untranslated region (3-UTR) of target mRNAs, leading to mRNA degradation or translational repression. However, under certain conditions, some miRNAs promote gene translation or transcription [10]. These small RNAs are key modulators of numerous biological pathways associated with obesity. In the liver, they play essential roles in metabolic processes, including lipogenesis, gluconeogenesis, and cholesterol homeostasis. Moreover, they are critical for development and hepatocyte differentiation, and actively contribute to the progression of liver fibrosis and intercellular communication by modulating responses to cellular injury and inflammation [11–14]. Aberrant miRNAs expression has also been associated with metabolic dysfunction as well as systemic complications, including insulin resistance, type 2 diabetes, cardiovascular disease, and MASLD [15–19]. Furthermore, miRNAs also serve as mediators of inter-organ communication, orchestrating coordinated metabolic responses across multiple organs [20], highlighting their biomarker and therapeutic potential [21].

Circulating miRNAs have emerged as promising biomarkers for overweight and obesity in adults, reflecting underlying metabolic and physiological alterations [22, 23]. Recent studies suggest that miRNAs may serve as biomarkers for subclinical vascular damage in patients with severe obesity and as useful tools for monitoring metabolic and vascular responses following bariatric surgery [23]. In liver tissue, some miRNAs such as hsa-miR-21, hsa-miR-128, hsa-miR-223, and hsa-let-7a, may be particularly relevant to the progression of obesity-related diseases, MASLD and insulin resistance (IR) [24, 25]. These miRNAs have been identified as critical regulators of metabolic processes, including adipogenesis, hepatic lipid metabolism, inflammation, and immune response [26–29], relevant aspects in the context of obesity. However, the specific expression patterns and clinical associations of these key hepatic miRNAs remain poorly defined; human data are scarce or controversial, and their relationships with clinical features of patients with obesity are largely unknown. This gap hampers our understanding of the molecular mechanisms underlying the pathogenesis of obesity and limits the development of personalized therapies [30, 31]. Identification of the role of specific miRNAs in obesity pathogenesis may favor the development of personalized therapeutic approaches that consider metabolic status and AMPs, such as hypertension or type 2 diabetes. These therapies can optimize treatment efficacy, reduce medication use, and ultimately improve patient outcomes [32, 33].

We thus hypothesized that obesity and AMPs may alter specific hepatic miRNAs, and our aim was to assess the expression levels of hsa-miR-21-3p, hsa-miR-128-3p, hsa-miR-223-3p, and hsa-let-7a in liver tissue from patients with obesity and to investigate the potential associations between these miRNAs and various clinical and demographic variables, including sex and obesity-related AMPs. Our results revealed altered expression of hsa-miR-128-3p and hsa-miR-223-3p in patients with obesity compared with healthy controls. These findings may provide further insights into the involvement of specific miRNAs in the pathophysiology of obesity and its AMPs.

## Materials and Methods

### Patients and Control Individuals

Our study sample included 54 patients with obesity, ranging from class II (body mass index [BMI] 35–39.9 kg/m²) to class V (BMI ≥ 60 kg/m²), who underwent laparoscopic sleeve gastrectomy at the [University Hospital of Salamanca] as well as 25 controls who underwent laparoscopic cholecystectomy. BMI categories were defined according to the International Federation for the Surgery of Obesity and Metabolic Disorders (IFSO) terminology [34].

Exclusion criteria for patients included previous bariatric metabolic surgery, total parenteral nutrition within the preceding six months, debilitating chronic diseases (e.g., malignancies), acute intercurrent conditions (e.g., infections), or the use of immunosuppressive drugs. Control subjects undergoing laparoscopic cholecystectomy were screened to exclude overt metabolic disease or other liver diseases unrelated to gallbladder disease. The same exclusion criteria were applied for the control group, in addition to BMI ≥ 30 kg/m² and malnutrition (BMI < 17 kg/m²). Body weight, height, and medical history were recorded for all participants. Additionally, fasting peripheral blood (PB) samples were collected before surgery for biochemical analyses, including fasting glucose levels and lipid profile assessments. Although not entirely disease-free, these controls enable access to liver tissue and are thoroughly phenotyped, allowing a structured evaluation of potential bias from gallstone disease or other metabolic factors. All samples were obtained after providing detailed information to participants and securing their written informed consent, in strict compliance with Spanish legal regulations for clinical research and the ethical guidelines established by the Ethics Committee of the [University Hospital of Salamanca].

### Sample Preparation and Processing

Intraoperative liver biopsies were obtained from segment III (left lateral segment) using a wedge technique. Each specimen had a capsular surface footprint of approximately 2.0 × 1.5 cm (length × width) and extended into the parenchyma as a wedge. To minimize confounding from surgical manipulation or hemodynamic changes, biopsies were collected immediately after induction of general anesthesia and before initiation of the planned surgical procedure. Tissue was immediately immersed in RNAlater, kept on ice during handling, and within 30 min was trimmed into slices ≤ 0.5 cm in thickness to ensure complete reagent penetration, aliquoted, and stored at − 80 °C until RNA extraction. Total RNA was extracted from liver tissue using the TRIzol method (Invitrogen), following the manufacturer’s recommendations. The extracted RNA was stored at -80 °C after determining its quantity and purity using a spectrophotometer, and the absorbance was measured at 260 nm and the 260/280 nm absorbance ratio.

### RNA Extraction, Reverse Transcription, and Quantitative Real-Time PCR (qRT-PCR)

For mRNA expression analysis, complementary DNA (cDNA) was synthesized from total RNA via reverse transcription using a High-Capacity cDNA Reverse Transcription Kit (Applied Biosystems), following the manufacturer’s instructions. qRT-PCR was performed using SYBR Green PCR Master Mix (Applied Biosystems) in conjunction with gene-specific primer sets. For miRNAs expression analysis, reverse transcription of total RNA-containing miRNAs was performed using a miRNA-specific primer-based system (miRCURY LNA Universal RT microRNA PCR kit, Exiqon). Subsequent qPCR was conducted using miRNA-specific primers (Table 1) and the ExiLENT SYBR Green Master Mix (Exiqon). All qRT-PCR reactions were duplicated on a StepOnePlus™ Real-Time PCR System (Applied Biosystems). Primer specificity was confirmed by melt curve analysis. The threshold cycle (Ct) values, that is, the number of cycles required to reach the fluorescence detection threshold, were determined for each target. The relative gene expression levels were calculated using the 2 − ΔΔCt method. The nucleotide sequences and annealing temperatures of the primer pairs used are listed in Table 1.

### Evaluation of Reference Genes for Normalization

To identify the most suitable endogenous controls for data normalization, the expression stability of the candidate reference genes was evaluated using three widely accepted algorithms: NormFinder [35], GeNorm [36], and BestKeeper [37]. NormFinder and GeNorm analyses were conducted using GenEX software (MultiD), whereas BestKeeper analysis was performed using Microsoft Excel-based tools [38]. Reference gene selection was performed independently for miRNA expression analyses, utilizing distinct miRNA candidate genes such as hsa-miR-152-3, hsa-miR-103a-3p, hsa-miR-24-3p, and U6 small nuclear RNA (Table 1). The target miRNAs hsa-miR-128-3p, hsa-miR-21-3p, hsa-let-7a-5p, and hsa-miR-223-3p were analyzed in this study (Table 1).

**Table 1:** Sequences of oligonucleotides used as primers for mRNA-specific RT-PCR analysis

miRNA	Sequence
Internal control miRNA candidates	
hsa-miR-152-3p	UCAGUGCAUGACAGAACUUGG
hsa-miR-103-3p	AGCAGCAUUGUACAGGGCUAUGA
hsa-miR-24-3p	UGGCUCAGUUCAGCAGGAACAG
hsa-miR-23b-3p	AUCACAUUGCCAGGGAUUACCAC
U6 (RNU6-1)	GUGCUCGCUUCGGCAGCACAUAUACUAAAAUUGGAACGAUACAGAGAAGAUUAGCAUGGCCCCUGCGCAAGGAUGACACGCAAAUUCGUGAAGCGUUCCAUAUUUU
**Target miRNAs**	
hsa-miR-128-3p	UCACAGUGAACCGGUCUCUUU
hsa-miR-21-3p	CAACACCAGUCGAUGGGCUGU
hsa-let-7a-5p	UGAGGUAGUAGGUUGUAUAGUU
hsa-miR-223-3p	UGUCAGUUUGUCAAAUACCCCA

### Histological Studies

For histological analysis, hematoxylin and eosin (HE) staining was employed [39]. A portion of each liver biopsy sample was fixed in 10% formalin and stained with HE and Masson’s trichrome for standard histopathological evaluation. MASLD was diagnosed using standard criteria, and the severity of the disease was established using the NAFLD activity score (NAS) described by Kleiner [40]. NAS scoring was applied based on histological assessment of steatosis (0–3), lobular inflammation (0–3), and hepatocellular ballooning (0–2), yielding a total activity score ranging from 0 to 8 [40]. Although the recent nomenclature recommends the use of MASLD/MASH, we applied the NAFLD Activity Score (NAS) because it remains the most widely accepted and validated histological scoring system for grading disease activity.

To explore potential associations between miRNA expression and histopathological severity, correlation analyses were conducted between the expression levels of miRNAs showing statistically significant differences between groups with or without obesity and the NAFLD Activity Score (NAS). The NAS and its components (steatosis grade, lobular inflammation, and hepatocellular ballooning) were analyzed independently, and statistical significance will be set at *p* < 0.05. Histological analyses were feasible in a subset of 46 patients with obesity (from the total cohort of 54), and all correlation analyses were performed on this subset.

### Statistical Analysis

Given the predominantly non-normal distribution of the data and the small sample size, comparisons were compared using the Mann–Whitney U test. Effect sizes were calculated using Cliff’s Delta (δ). Raw p-values (p_raw) were obtained using the Mann–Whitney U test. Bonferroni correction and false discovery rate control using the Benjamini–Hochberg procedure were applied within each predefined block of analyses, with adjusted significance thresholds calculated according to the number of tests performed in each block. Categorical variables were reported as absolute and relative frequencies and were analysed using the chi-square (χ²) test or Fisher’s exact test when the expected frequencies were less than five. Correlations between quantitative variables were assessed using Pearson’s correlation coefficient (r) or Spearman’s rank correlation coefficient. A two-sided p-value < 0.05 was considered statistically significant.

A multivariable logistic regression model was applied, with obesity versus control as the dependent variable, to assess whether has-miR-128-3p and has-miR-223-3p expression were independently associated with obesity after adjustment for age, sex, diabetes, DL, and HT). A backward stepwise procedure was used to select variables, and the final model was verified by fitting a non-stepwise model including the same covariates. A two-sided P value < 0.05 was considered statistically significant. All analyses were performed using IBM SPSS Statistics, version 30.0.

Discrimination for miRNAs predicting obesity was summarized by the area under the ROC curve (AUC) computed from model-predicted probabilities. We fit logistic regression models with individual miRNA levels and a two-marker model as predictors of the binary outcome (1 obese, 0 control). Internal validation used 200 bootstrap samples; in each replicate, the model was refitted, AUCs were computed in the bootstrap sample and in the original dataset, optimism was defined as their difference, and the mean optimism was subtracted from the apparent AUC to obtain the optimism-corrected AUC. Replicates with a single outcome class were discarded. Analyses were conducted in Python (scikit-learn) with a fixed random seed.

## Results

### Analysis of the Basal Characteristics of Individuals Included

Our study included 54 patients with obesity (41 women and 13 men), with a mean age of 45.28 (SEM = 1,52) years; a body weight of 130.75 (3.14) Kg; and BMI of 49.33 (1.02) kg/m^2^. In addition, 25 individuals without obesity (14 women and 11 men) were included with a mean age of 55.92 (3.41) years, body weight of 68. 91 (2.78) Kg, and a BMI of 24.41 (0.78) kg/m2. The baseline characteristics of the individuals included in the study and the comparison between individuals with and without obesity are summarised in Table 2. Patients with obesity had a higher BMI, body weight, and a higher prevalence of diseases associated with obesity, such as diabetes, arterial hypertension (HTN), and dyslipidemia (DL).

**Table 2:** Basal characteristics of the individuals with or without obesity

Variable	Individuals with obesity (*n* = 54)	Individuals without obesity (*n* = 25)	*p*
Age (years)	45.28 (1.52)	55.92 (3.41)	0.007
Male(n)/Female (n)	13/41	11/14	0.673
Body weight (kg)	130.75 (3.14)	68.91 (2.78)	< 0.001
BMI (kg/m2)	49.33 (1.02)	26.41 (0.78)	< 0.001
Fasting blood glucose (mg/ dL)	110.7 (5.88)	95.57 (2.69)	0.193
Total cholesterol (mg/dL)	194.45 (5.1)	199.89 (9.25)	0.619
Triglycerides (mg/dL)	144.79 (11.52)	111.79 (9.51)	0.114
LDL (mg/ dL)	114.09 (5.33)	124.22 (8.21)	0.360
HDL (mg/dL)	46.92 (1.67)	53.5 (3.75)	0.181
ALP (IU/L)	73.15 (2.45)	86.91 (7.47)	0.112
GGT (IU/L)	32.94 (3.03)	45.59 (6.9)	0.141
AST (IU/L)	23.92 (1.67)	28.65 (4.83)	0.448
ALT (IU/L)	30.74 (2.29)	32.47 (3.95)	0.764
HbA1c	6.35 (1.47)	6.14 (1.07) *	0.904
Diabetes [n (%)]	14 (25.96%)	2 (8%)	< 0.001
HTN [n (%)]	26 (48.15%)	8 (32%)	< 0.001
DL [n (%)]	15 (27.78%)	2 (8%)	< 0.001

Quantitative variables are presented as mean (standard deviation), and qualitative variables as n (%)

Body mass index (BMI), arterial hypertension (HTN), dyslipidemia (DL). Low-density lipoprotein (LDL), high-density lipoprotein (HDL), alkaline phosphatase (ALP), gamma-glutamyl transferase (GGT), aspartate aminotransferase (AST), alanine aminotransferase (ALT), and glycated hemoglobin (HbA1c)

* The number of controls (n) is 7

### Expression of Different miRNAs in Liver Tissue

**Fig. 1:**
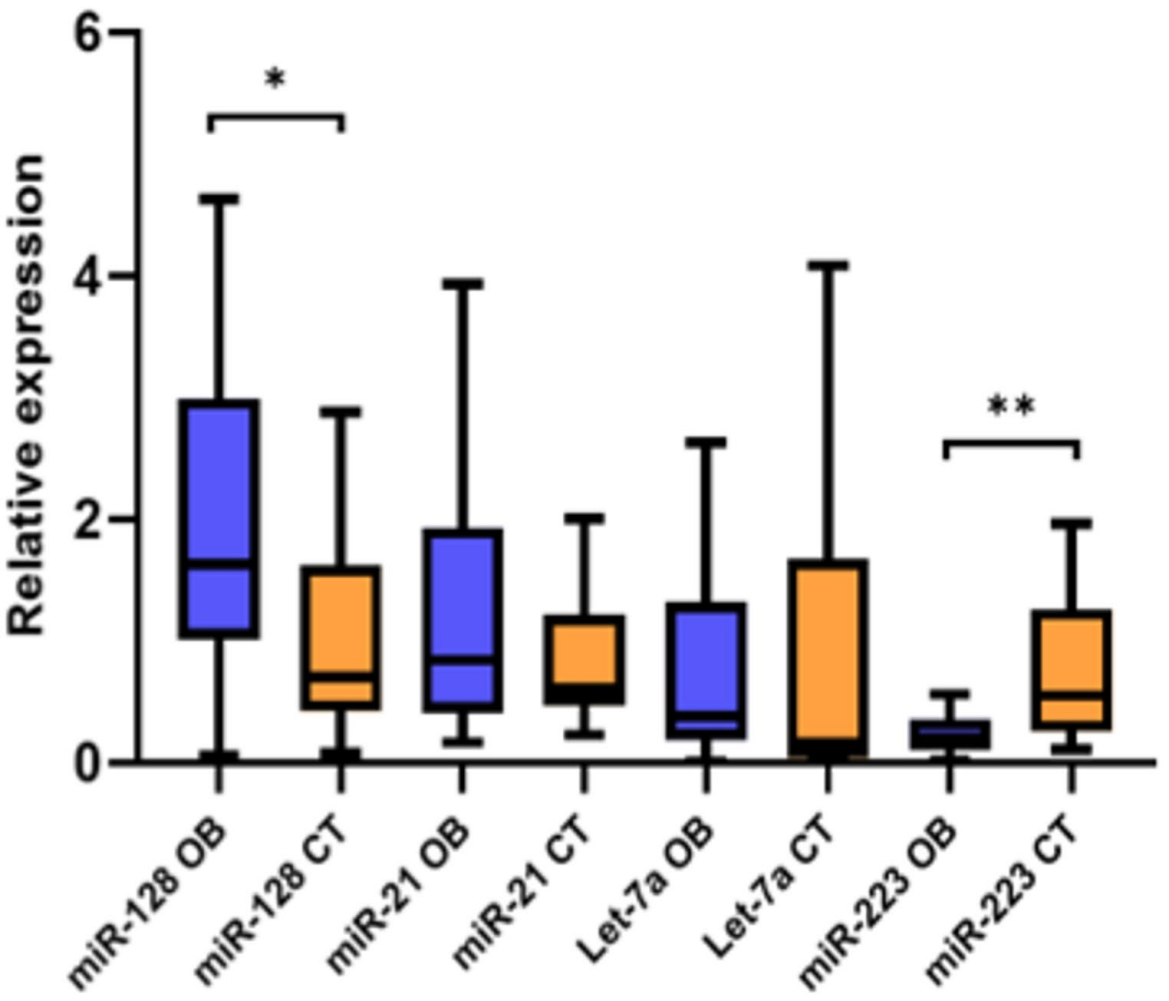
Relative expression levels of selected miRNAs in patients with obesity and individuals without obesity. The analysis included four miRNAs: hsa-miR-21-3p, hsa-miR-128-3p, hsa-let-7a-5p, and hsa-miR-223-3p in patients with obesity (OB) and individuals without obesity (CT). Statistical comparisons were performed using the Mann–Whitney U test. Boxplots show the median (center line), interquartile range (box), and whiskers extending to 1.5× the interquartile range (Tukey method). *P* < 0.05 was considered statistically significant; *p* < 0.05 (*), *p* < 0.01 (**). Patients with obesity: *n* = 54; Individuals without obesity: *n* = 25

After analyzing our own samples, we observed that hsa-miR-152-3p, hsa-miR-103a-3p, and hsa-miR-24-3p were stable. However, the stability indices of hsa-miR-24-3p were consistent across all three algorithms (0.61, 0.70, and 0.81), whereas other candidates (e.g., hsa-miR-152-3p and hsa-miR-103a-3p) showed lower values in two algorithms but greater variability in the third (Supplementary Table 1). This consistency across methods supports the selection of hsa-miR-24-3p as the most reliable internal control. qPCR analysis confirmed that hsa-miR-24-3p was consistently and specifically amplified in both controls and patients with obesity (Supplementary Tables 2 and Supplementary Figs. 1 and 2). Thus, this miRNA was used to normalize the expression levels of hsa-miR-21-3p, hsa-miR-128-3p, hsa-let-7a-5p, and hsa-miR-223-3p. Figure 1 presents a comparative analysis of the expression of different miRNAs in individuals with and without obesity. The results showed significantly higher expression of hsa-miR-128-3p in the group with obesity than in the non-obesity group (*p* = 0.003), while hsa-miR-223-3p expression was decreased compared to the control group (*p* = 0.001) (Fig. 1).

No significant differences were found in hsa-miR-21-3p and hsa-let-7a-5p expression in liver tissue between the obesity and control groups. Although this study is exploratory, we applied Bonferroni correction, and the main results remained consistent with the unadjusted analyses. Data about the expression of hsa-miR-128-3p and hsa-miR-223-3p remained significant after Bonferroni correction (adjusted *p* = 0.0125) (Supplementary Table 3) and survived FDR adjustment. Cliff’s δ values for hsa-miR-128-3p and hsa-miR-223-3p were 0.42 and 0.49, respectively, indicating their potential biological relevance (Supplementary Table 4).

In addition, hsa-miR-128-3p and hsa-miR-223-3p showed moderate discriminative capacity for obesity (Supplementary Figs. 3 A and 3B). For hsa-miR-128-3p, the apparent AUC was 0.715 and the optimism-corrected AUC was 0.719. For hsa-miR-223-3p, the apparent AUC was 0.764 and the optimism-corrected AUC was 0.743. A combined two-marker model performed better, with an apparent AUC of 0.840 and an optimism-corrected AUC of 0.826.

### Sex Influence on the Expression of the Studied miRNAs

**Fig. 2:**
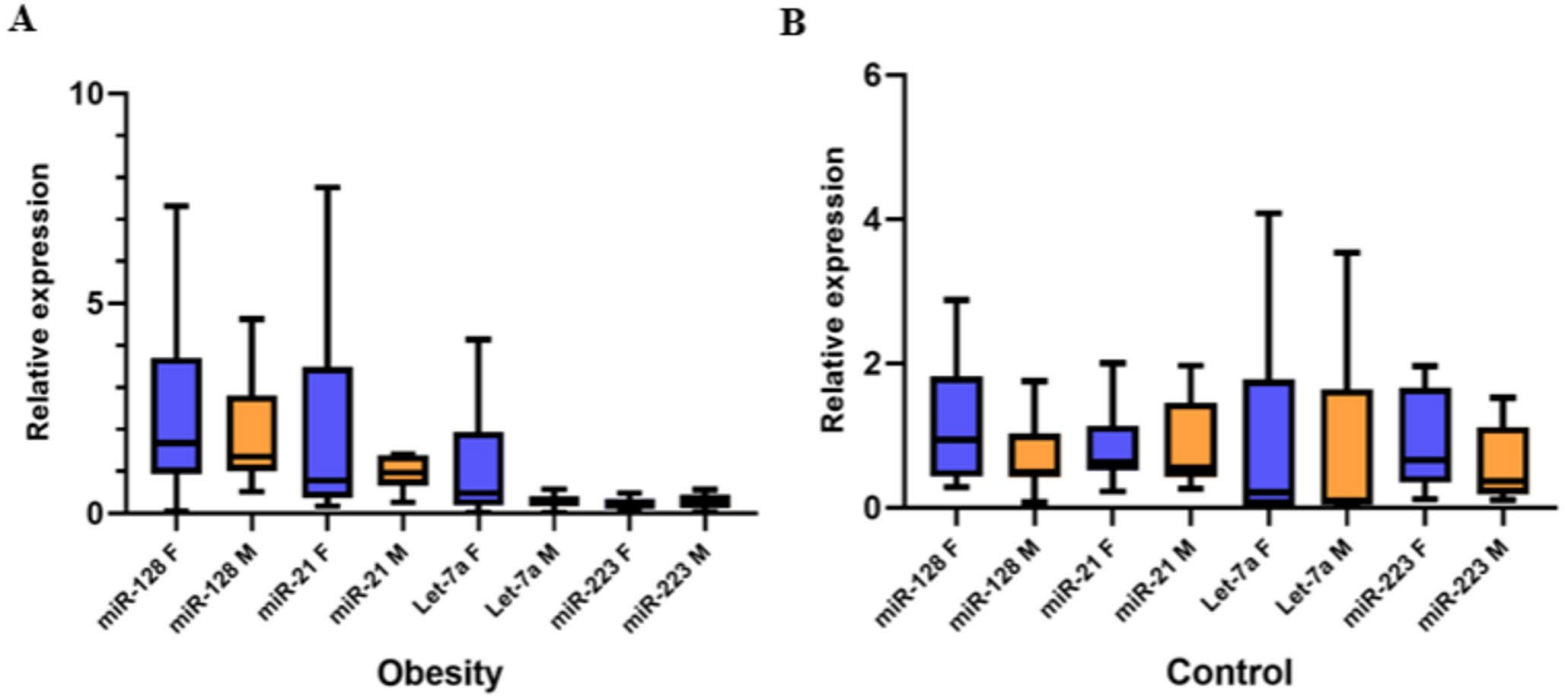
Relative expression of miRNAs in patients with obesity and healthy controls stratified by sex. The relative expression levels of hsa-miR-21-3p, hsa-miR-128-3p, hsa-let-7a, and hsa-miR-223-3p were evaluated in individuals with obesity and healthy controls, stratified by sex. (**A**) The relative expression levels of hsa-miR-21-3p, hsa-miR-128-3p, hsa-let-7a, and hsa-miR-223-3p were evaluated in female (F) patients vs. male (M) patients with obesity. (**B**) The relative expression levels of hsa-miR-21-3p, hsa-miR-128-3p, hsa-let-7a, and hsa-miR-223-3p were evaluated in female (F) vs. male (M) controls. The patient group with obesity included 41 women (F) and 13 men (M), while the control individuals included 14 women and 11 men. Group comparisons were performed using the Mann–Whitney U test. Boxplots show the median (center line), interquartile range (box), and whiskers extending to 1.5× the interquartile range (Tukey method)

Because the expression of certain miRNAs may be influenced by sex, their relative expression was analyzed in patients with obesity and healthy controls, stratified by sex (Fig. 2). Although no statistically significant differences were found in the expression levels of these miRNAs between women and men, a trend toward higher expression levels in women with obesity was observed.

### Expression of miRNAs in Patients with Obesity, Stratified by the Presence of Obesity-AMPs

**Fig. 3:**
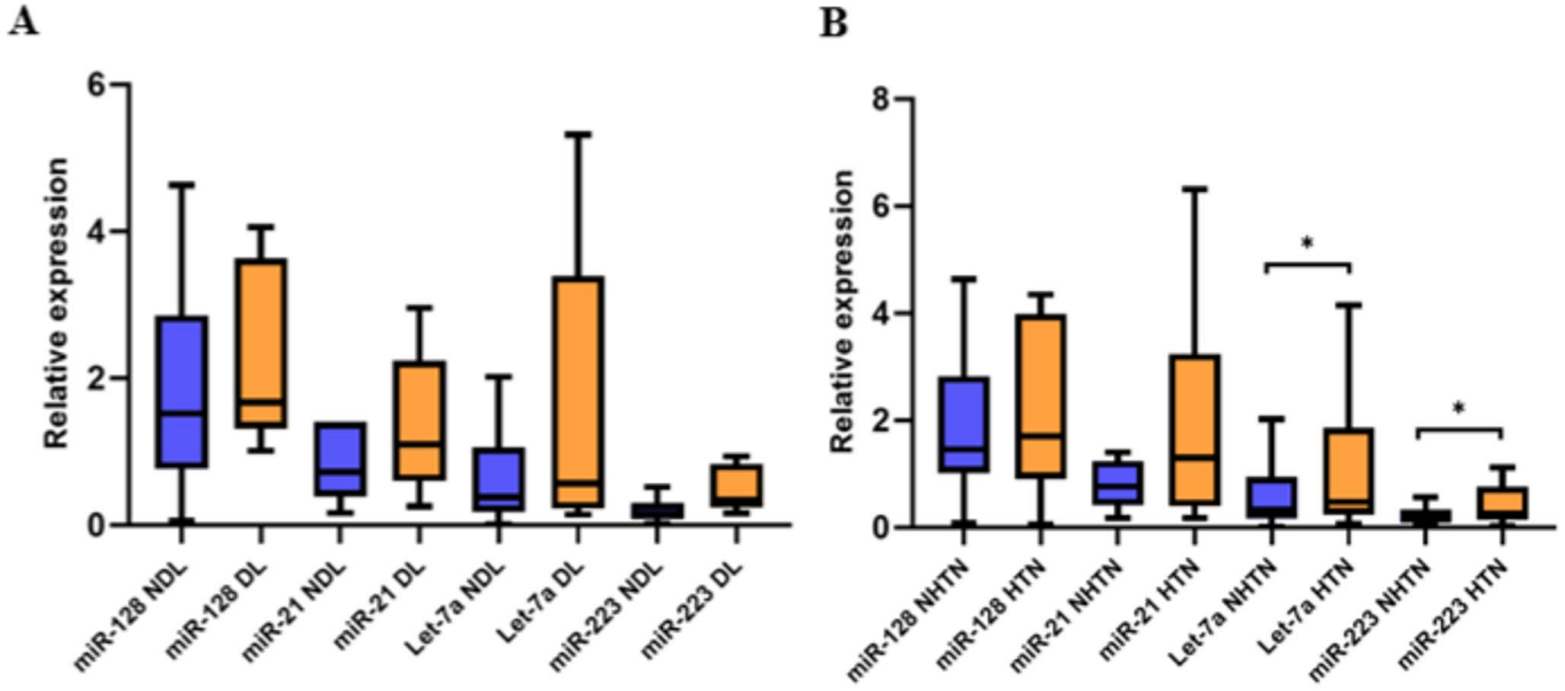
Relative expression of miRNAs in patients with obesity stratified by dyslipidemia (DL) and hypertension (HTN) status. (**A**) Relative expression of hsa-miR-128-3p, hsa-miR-21-3p, hsa-let-7a-5p, and hsa-miR-223-3p in patients with obesity with DL (OB-DL) (*n* = 15) and without DL (OB-NDL) (*n* = 39). (**B**) Relative expressions of hsa-miR-128-3p, hsa-miR-21-3p, hsa-let-7a-5p, and hsa-miR-223-3p in patients with obesity and HTN (OB-HTN) (*n* = 26) and without HTN (OB-NHTN) (*n* = 28). Group comparisons were performed using the Mann–Whitney U test. Boxplots show the median (center line), interquartile range (box), and whiskers extending to 1.5× the interquartile range (Tukey method). *p ≤ 0.05

The expression of miRNAs was evaluated in patients with obesity and various AMPs, including type 2 diabetes, DL, and HTN, to determine whether these conditions influence the expression profiles of these miRNAs. When comparing patients with DL to those without, no statistically significant differences in miRNA expression were detected. However, a consistent trend toward higher expression levels, particularly for hsa-miR-128-3p and hsa-miR-21-3p, was observed in those with obesity and DL (Fig. 3A). Of note, as shown in Fig. 3B, the expression of hsa-let-7a was significantly increased in patients with obesity and HTN compared to those with obesity but without HTN (*p* = 0.026) (Fig. 3B). Additionally, a significant upregulation of hsa-miR-223-3p was observed in patients with obesity and HTN compared to that in the control group (*p* = 0.026). However, neither of these comparisons was significant after Bonferroni correction nor they did survive FDR correction.

### Multivariable Logistic Regression Analysis of miRNAs in Patients with Obesity

After logistic regression analysis, expression of hsa-miR-128-3p (OR 2.62, 95% CI 1.26 to 5.45; *p* = 0.01) and hsa-miR-223-3p (OR 0.02, 95% CI 0.00 to 0.34; *p* = 0.01) retained statistical significance. Other variables statistically significant in the model were age (OR 0.92, 95% CI 0.86 to 0.98; *p* = 0.01) and DL (OR 78.40, 95% CI 1.48 to 4141.55; *p* = 0.03) (Supplementary Table 5). In this model, older age and higher hsa-miR-223-3p levels were associated with a lower probability of obesity, whereas DL and increased hsa-miR-128-3p expression were associated with a higher probability of obesity. Sex, HTN, and diabetes did not reach statistical significance.

### Associations Between miRNAs Expression and Histological Features of NAFLD in Patients with Obesity

**Fig. 4:**
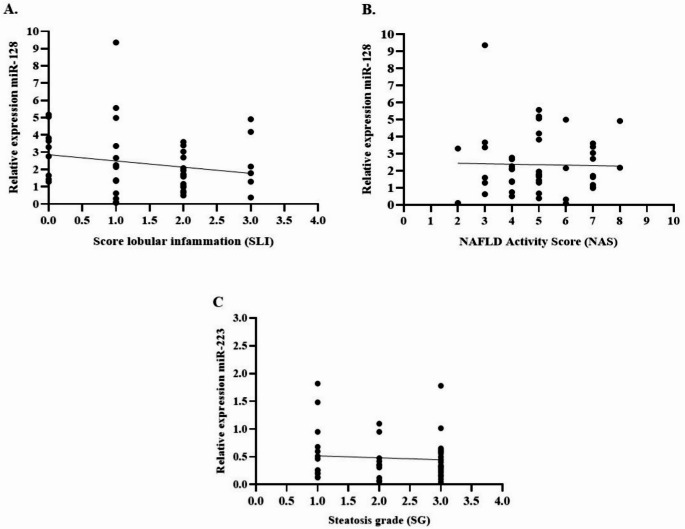
Relative expression levels of hsa-miR-128-3p and hsa-miR-223-3p with histological features of NAFLD in patients with obesity. (**A**) Correlation between relative expression of hsa-miRNA-128-3p in hepatic tissue and lobular inflammation (SLI) (ρ = − 0.190; *p* = 0.270). (**B**) Correlation between hsa-miR-128-3p expression and the NAFLD Activity Score (NAS) (ρ = − 0.023; *p* = 0.881). (**C**) Correlation between the relative expression of hsa-miR-223-3p and steatosis grade (SG) (ρ = − 0.075; *p* = 0.90). Correlations were assessed using Spearman’s rank correlation coefficient. Statistical significance was set at *p < 0.05. Patients with obesity (*n* = 46) represent the subset of the total cohort (*n* = 54) for whom histological analyses were possible

 We examined the potential association between the expression levels of hsa-miR-128-3p and hsa-miR-223-3p, the two miRNAs that exhibited significant differential expression in patients with obesity compared with controls, and the histological features of liver injury as assessed by the total NAS. No significant correlations were found between miRNA expression and total NAS, lobular inflammation, or hepatic steatosis for either hsa-miR-128-3p or hsa-miR-223-3p (Fig. 4).

## Discussion

In this study, we found that hepatic hsa-miR-128-3p was significantly upregulated in patients with obesity, whereas hsa-miR-223-3p was markedly downregulated compared to controls without obesity. A careful normalization strategy was implemented before expression analysis, and hsa-miR-24-3p emerged as the most stable across multiple algorithms (Table 1), which is consistent with previous findings in liver tissue [41], supporting its suitability as an internal control.

Elevated hepatic miR-128-3p in individuals with obesity is consistent with previous studies in humans and mice showing that miR-128 regulates insulin receptor (INS-R) expression in visceral adipose tissue; its upregulation induces INS-R downregulation and contributes to early insulin resistance in obesity [42]. In human liver tissue, hsa-miR-128 has been experimentally validated to target key regulators of cholesterol metabolism, including low-density lipoprotein receptor (LDLR) and ATP-binding cassette transporter A1 (ABCA1), thereby modulating lipid uptake and efflux. Additionally, hepatic expression profiles and genetic association studies have linked miR-128 to DL and abnormal blood lipid profiles, supporting its involvement in lipid homeostasis [43, 44]. Consistent with human findings, murine overexpression of mmu-miR-128 results in altered hepatic lipid-trafficking proteins and elevated circulating cholesterol and triglyceride levels [44]. Taken together, these mechanistic insights suggest that hsa-miR-128-3p contributes to obesity-related liver pathology by impairing insulin signaling and promoting lipid accumulation, both of which are central to the progression of metabolic dysfunction-associated steatotic liver disease (MASLD).

Beyond its role in cholesterol metabolism and insulin signaling, hsa-miR-128-3p has been experimentally validated as a master regulator with pro-inflammatory and pro-oxidative properties in the context of obesity and NAFLD, primarily by directly targeting the silent information regulator sirtuin 1 (SIRT1) [45, 46]. Repression of SIRT1 disrupts the activation of Forkhead box O (FOXO) transcription factors, leading to a reduced antioxidant response and the accumulation of reactive oxygen species (ROS) [47]. This oxidative stress is further intensified by activation of the NLRP3 inflammasome, as both SIRT1 and FOXO3 serve as negative regulators of NLRP3-mediated inflammation [48, 49]. In parallel, hsa-miR-128-3p interferes with lipid metabolism by suppressing the peroxisome proliferator-activated receptor gamma (PPAR-γ), contributing to adipose and hepatic tissue dysfunction, hepatic lipid accumulation, and the progression from obesity to metabolic dysfunction–associated steatotic liver disease (MASLD) and hepatic steatosis [50, 51].

In contrast, hepatic hsa-miR-223-3p expression was significantly reduced in patients with obesity, consistent with its established role as a regulator of liver homeostasis. Experimentally validated targets of this miRNA include genes involved in lipid and cholesterol metabolism, apoptosis, cell proliferation, and inflammatory signaling. Accordingly, hsa-miR-223-3p regulates the expression of ABCA1 (via the transcription factor SP3), as well as 3-hydroxy-3-methylglutaryl-CoA synthase 1 (HMGCS1) [52]. For instance, its downregulation leads to disrupting cholesterol efflux and hepatic lipid accumulation. In addition, miR-223-3p suppresses Absent in melanoma 2 (AIM2)-dependent IL-1β production in Kupffer cells, thereby exerting potent anti-inflammatory effects [53, 54]. In murine models, loss of miR-223 (miR-223⁻/⁻) leads to dysregulated expression of hepatic lipid genes and disruption of cholesterol homeostasis [52], while its overexpression negatively regulates diacylglycerol lipase alpha (DAGLA), impacting lipid turnover [25]. Therefore, downregulation of hsa-miR-223-3p in patients with obesity may amplify obesity-related systemic inflammatory responses, impair lipid metabolism, and promote the progression of obesity-related liver pathology, including MASLD.

Notably, functional studies have shown that genetic ablation of miR-223 exacerbates liver injury, suggesting a protective role of this miRNA in hepatic pathology. Mechanistically, miR-223 directly suppresses IL-6 expression in neutrophils, thereby attenuating pro-inflammatory IL-6 signaling and mitigating hepatic damage in models of ALD [55]. Moreover, the loss of miR-223 leads to upregulation of the PDZ-binding motif (TAZ), a transcriptional co-activator implicated in promoting fibrosis during metabolic dysfunction-associated steatohepatitis (MASH) progression [56]. Together, these mechanistic findings highlight that downregulation of hsa-miR-223 may disrupt the fine-tuned control of inflammatory and fibrotic signaling in the liver. Consistent with this, previous studies have shown that hepatic miRNAs, including hsa-miR-128 and hsa-miR-223, are altered in liver diseases and regulate key processes such as lipid metabolism, inflammation, and fibrosis [25, 57–59].

Importantly, the hepatic expression levels of both hsa-miR-128-3p and hsa-miR-223-3p remained significantly different from controls after Bonferroni correction in patients with obesity (Supplementary Table 2). Additionally, both miRNAs showed Cliff’s δ values of 0.40 and 0.56, respectively (Supplementary Table 3), indicating moderate-to-large effect sizes and potential biological relevance. These findings along with the results of the multivariable analysis suggest that these miRNAs may play a role in obesity-related metabolic dysfunction and highlight their potential relevance as exploratory biomarkers or therapeutic candidates, particularly given that their hepatic expression patterns in bariatric surgery patients had not been previously characterized.

Notably, our findings are novel and provide new insights into liver-specific miRNA regulation in patients with obesity. A recent meta-analysis reviewed 17 studies on miRNA changes after bariatric surgery and identified 14 consistently modulated miRNAs [60]. Neither hsa-miR-128-3p nor hsa-miR-223-3p was among them, highlighting the potential relevance of our observations. It is also noteworthy that most studies conducted to date have been based on blood, adipose tissue, and skeletal muscle [60, 61], while those that have focused on liver tissue have neither been conducted in the clinical context of bariatric surgery nor examined the specific clinical correlations with AMPs [61, 62]. Therefore, our study represents an important contribution to filling this gap, given that the liver plays a central role in obesity-related metabolic dysfunction and its status as a key target of bariatric surgery. Ideally, longitudinal studies following bariatric surgery could provide insights into their dynamics during metabolic recovery. Future research should validate these findings in larger cohorts, assess whether circulating levels may serve as minimally invasive biomarkers, and investigate mechanistic roles in preclinical models, thereby helping to bridge the gap between expression data and clinical relevance.

Given their involvement in liver pathology, we next assessed whether these miRNAs correlated with histological markers of liver injury, as measured by NAS score (*n* = 46). No statistically significant correlations were observed for hsa-miR-128-3p and hsa-miR-223-3p with the total NAS or any NAS component. These results indicate that, within our cohort, the expression of these miRNAs is not directly associated with the histological severity of liver damage.

Although hsa-miR-223-3p could be potentially linked to MASLD progression, considering its aforementioned potential effects, this lack of association found in our study supports the hypothesis that the dysregulation of these miRNAs may be more closely related to systemic obesity-associated mechanisms rather than to the histological severity of liver damage per se. However, we recommend that future larger studies account for potential confounders, such as age and BMI, using partial correlation or multivariable regression to strengthen the assessment of these associations.

Regarding sex differences, several studies have reported that some of the miRNAs selected in this study exhibit sex-specific expression or are regulated by sex hormones in various tissues, including the liver [63–67]. In our cohort, women with obesity tended to display higher hepatic levels of these miRNAs, however, these differences did not reach statistical significance (Fig. 2). Although previous studies have suggested that miRNAs, such as hsa-let-7a and hsa-miR-221, display a female-specific elevation in metabolic syndrome [63]. In murine models, mmu-let-7a can regulate glucose metabolism through the suppression of the insulin-PI3K-mTOR signaling pathway [68], while mmu-miR-221-3p exacerbates obesity-induced IR by targeting SOCS1 (Suppressor Of Cytokine Signaling 1) in adipose tissue [69].

Exploring the influence of AMPs, such as HTN and DL, on the expression of this group of miRNAs in patients with obesity, we found that patients with obesity and HNT exhibited a significant hepatic overexpression of hsa-let-7a-5p and hsa-miR-223-3p compared to patients with obesity without hypertension (Fig. 3). Notably, when applying the Bonferroni-adjusted threshold, the p-values for these miRNAs were very close to this threshold, suggesting that the initially observed significance could be supported. These observations align with reports of hsa-let-7a-5p role in the pathogenesis of essential hypertension and its potential as a companion diagnostic marker [70]. Importantly, mmu-let-7a-5p acts as a negative regulator of cardiac inflammation and fibrosis by suppressing the expression of interleukin-6 and collagen gene, which may represent a new potential therapeutic target for treating hypertensive cardiac fibrosis [71]. Similarly, mmu-miR-223 has been linked to various cardiovascular pathologies, acting as a therapeutic target and biomarker, and its upregulation alleviates pulmonary arterial hypertension in murine models [72]. To our knowledge, this is the first study connecting hepatic hsa-miR-223-3p to HTN, underscoring the need for further work to confirm these links and clarify underlying mechanisms. It is important to note that although a statistically significant increase in these proteins was not found in patients with DL, some studies have demonstrated the upregulation of mmu-miR-128-3p in hypercholesterolemic mice. Inhibition of this miRNA leads to the downregulation of crucial genes involved in cholesterol metabolism and, consequently, a reduction in serum cholesterol, which has led to suggestions for its potential therapeutic implications in reversing hypercholesterolemia [73].

Although our study found that hsa-miR-21-3p expression showed a non-significant trend toward upregulation in patients with obesity, as well as in those with AMPs, it is noteworthy that in patients with obesity and HTN, the unadjusted p-value of 0.065 was close to the Bonferroni-adjusted threshold (Supplementary Table 3), suggesting that this miRNA could have relevance in obesity-related AMPs. In fact, some reports have linked this miRNA to obesity and its associated conditions, including systemic hypertension and cardiac pathologies [74], and it has been proposed as a potential therapeutic tool to modulate metabolic dysfunction and control obesity [75]. Moreover, its levels have been positively correlated with serum lipid concentrations, suggesting its participation in altered cholesterol metabolism and possible uses as biomarkers of DL severity [76].

The main strengths of our study include the use of a reference control previously validated in our own samples, the homogeneity of the patient cohort, and the detailed characterization of our sample, including comprehensive histopathological analysis. In addition, the direction and effect size of the associations for hsa-miR-128-3p and hsa-miR-223-3p were consistent in both univariate and multivariable analyses. Although the limited sample size and the wide confidence interval observed for DL suggest that the multivariable model should be interpreted with caution. In this regard, one of the major limitations of our manuscript is the relatively small, single-center cohort, which may reduce statistical power, especially in sex- and AMPs-stratified analyses, and limits generalizability. Future studies with larger, multicenter cohorts will be necessary to validate and extend these results. In addition, the use of patients undergoing laparoscopic cholecystectomy as controls remains a key limitation. Although rigorous screening excluded overt liver disease, the inclusion of healthy controls in future studies could help confirm these results.

Nonetheless, our findings provide valuable insights into hepatic miRNA regulation in obesity and are consistent with prior mechanistic studies. To establish temporal and causal relationships, future prospective longitudinal and interventional studies will be essential. Additionally, leveraging liver tissue obtained during bariatric surgery offers an opportunity to characterize obesity-related molecular changes directly in the liver—an approach not feasible in non-surgical cohorts. This unique framework enhances the novelty and translational relevance of our study, providing a model for future investigations into the molecular mechanisms of obesity-related liver disease.

Moreover, no functional experiments or direct target validation were performed to confirm the mechanistic implications of the observed miRNA changes; nonetheless, the proposed relationships are supported by previous reports. Regarding normalization, the use of a single reference gene in our qPCR analyses could introduce systematic error and reduce the reliability of relative quantification. The use of technical duplicates rather than triplicates is also a potential limitation. However, its targeted selection for hepatic miRNA studies in obesity provides a context-specific approach compared with studies relying on reference genes validated in other tissues or disease settings. Thus, we consider that our approach provides informative insights, while future studies should further strengthen robustness and reproducibility by validating reference gene stability or incorporating additional reference genes.

Collectively, these findings therefore provide a solid rationale for future research integrating functional assays and direct target validation, which will enable a more definitive understanding of the role of these miRNAs in obesity-related liver pathology and may support the development of targeted therapeutic strategies.

## Conclusions

This study provides novel evidence that miRNAs, such as hsa-miR-128-3p and hsa-miR-223-3p, are differentially expressed in the hepatic tissue of patients with obesity and may contribute to the molecular mechanisms underlying metabolic dysfunction. The associations identified with hypertension and the observed sex-specific expression trends suggest new avenues for investigating the potential role of these miRNAs as exploratory biomarkers and potential therapeutic candidates in the context of personalized medicine. Unlike previous studies that primarily examined circulating or adipose tissue-derived miRNAs, our study emphasizes the relevance of hepatic-specific miRNA expression profiling, offering a more direct understanding of the local regulatory processes in the liver. Overall, the data provide valuable insights into hepatic miRNA regulation in obesity and are consistent with prior mechanistic studies; however, they should be interpreted cautiously due to the small sample size. Larger, multicenter studies will be necessary to confirm these observations, robustly evaluate subgroup-specific effects, clarify the mechanistic roles of hepatic hsa-miR-128-3p and hsa-miR-223-3p, and further assess their potential relevance as exploratory biomarkers or therapeutic candidates in the context of obesity.

## Supplementary Information

Below is the link to the electronic supplementary material.

Supplementary Material 1

## Data Availability

No datasets were generated or analysed during the current study.
