# Supplementary material for: miR-128-3p and miR-223-3p as Potential Biomarkers of Metabolic Dysfunction in Liver Tissue from Patients Undergoing Metabolic Bariatric Surgery
Source: Obes Surg. 2026 Mar 12;36(4):1469–83. doi: 10.1007/s11695-026-08499-3 (PMC13083441; doi:10.1007/s11695-026-08499-3)
Supplement: Supplementary file 1 — Supplementary Material 1 [file 11695_2026_8499_MOESM1_ESM.doc]

**Supplementary Table 1.** Evaluation and normalization of reference genes using BestKeeper. NormFinder and GeNorm algorithms

| **miRNA** | **Algorithms** | | |
| --- | --- | --- | --- |
|  | **geNorm** | **BestKeeper** | **NormFinder** |
| hsa-miR-152-3p | 0.72 | 0.70 | 0.49 |
| **hsa-miR-24-3p** | **0.83** | **0.70** | **0.61** |
| hsa-miR-103a-3p | 0.72 | 0.80 | 0.41 |
| hsa-miR-23b-3p | 0.89 | 0.84 | 0.95 |
| U6 (RNU6-1) | 1.12 | 1.50 | 1.97 |

**Supplementary Table 2**. Per-sample Cq values of miR-124-3p

| Sample Name | Target Name | Cт | Cт Mean | Cт SD |
| --- | --- | --- | --- | --- |
| OB1 | miR- 24-3p | 24.26362 | 24.35796 | 0.133424 |
| OB1 | miR- 24-3p | 24.45231 | 24.35796 | 0.133424 |
| OB3 | miR- 24-3p | 22.32882 | 22.35386 | 0.035414 |
| OB3 | miR- 24-3p | 22.3789 | 22.35386 | 0.035414 |
| OB 5 | miR- 24-3p | 24.78122 | 24.5055 | 0.389922 |
| OB 5 | miR- 24-3p | 24.22978 | 24.5055 | 0.389922 |
| OB 7 | miR- 24-3p | 23.5076 | 23.67538 | 0.237269 |
| OB 7 | miR- 24-3p | 23.84315 | 23.67538 | 0.237269 |
| OB 9 | miR- 24-3p | 24.23217 | 24.85622 | 0.882542 |
| OB 9 | miR- 24-3p | 25.48027 | 24.85622 | 0.882542 |
| OB 11 | miR- 24-3p | 25.84751 | 25.77696 | 0.099762 |
| OB 11 | miR- 24-3p | 25.70642 | 25.77696 | 0.099762 |
| OB 13 | miR- 24-3p | 23.48869 | 23.63382 | 0.205241 |
| OB 13 | miR- 24-3p | 23.77894 | 23.63382 | 0.205241 |
| OB15 | miR- 24-3p | 29.25817 | 28.99306 | 0.374928 |
| OB15 | miR- 24-3p | 28.72795 | 28.99306 | 0.374928 |
| OB 17 | miR- 24-3p | 28.00651 | 28.38124 | 0.529948 |
| OB 17 | miR- 24-3p | 28.75597 | 28.38124 | 0.529948 |
| OB 19 | miR- 24-3p | 25.31243 | 24.87507 | 0.618524 |
| OB 19 | miR- 24-3p | 24.4377 | 24.87507 | 0.618524 |
| OB 21 | miR- 24-3p | 25.9549 | 25.57182 | 0.541755 |
| OB 21 | miR- 24-3p | 25.18875 | 25.57182 | 0.541755 |
| OB 23 | miR- 24-3p | 26.99228 | 26.51241 | 0.678638 |
| OB 23 | miR- 24-3p | 26.03254 | 26.51241 | 0.678638 |
| OB 25 | miR- 24-3p | 27.12246 | 27.0085 | 0.161155 |
| OB 25 | miR- 24-3p | 26.89455 | 27.0085 | 0.161155 |
| OB 27 | miR- 24-3p | 24.82274 | 24.2355 | 0.830482 |
| OB 27 | miR- 24-3p | 23.64826 | 24.2355 | 0.830482 |
| OB 29 | miR- 24-3p | 25.59915 | 26.07625 | 0.67472 |
| OB 29 | miR- 24-3p | 26.55335 | 26.07625 | 0.67472 |
| OB 31 | miR- 24-3p | 24.74117 | 24.65531 | 0.121429 |
| OB 31 | miR- 24-3p | 24.56944 | 24.65531 | 0.121429 |
| OB 33 | miR- 24-3p | 23.17335 | 23.23533 | 0.087656 |
| OB 33 | miR- 24-3p | 23.29732 | 23.23533 | 0.087656 |
| OB 35 | miR- 24-3p | 24.7596 | 25.33798 | 0.817955 |
| OB 35 | miR- 24-3p | 25.91636 | 25.33798 | 0.817955 |
| OB 37 | miR- 24-3p | 23.93755 | 23.3805 | 0.787793 |
| OB 37 | miR- 24-3p | 22.82345 | 23.3805 | 0.787793 |
| OB 39 | miR- 24-3p | 26.33046 | 26.28876 | 0.05896 |
| OB 39 | miR- 24-3p | 26.24707 | 26.28876 | 0.05896 |
| OB 41 | miR- 24-3p | 24.95885 | 24.25318 | 0.997959 |
| OB 41 | miR- 24-3p | 23.54752 | 24.25318 | 0.997959 |
| OB 45 | miR- 24-3p | 23.74546 | 23.43467 | 0.439522 |
| OB 45 | miR- 24-3p | 23.12388 | 23.43467 | 0.439522 |
| OB 47 | miR- 24-3p | 24.45468 | 24.353 | 0.143794 |
| OB 47 | miR- 24-3p | 24.25132 | 24.353 | 0.143794 |
| OB 49 | miR- 24-3p | 22.67846 | 22.61428 | 0.090758 |
| OB 49 | miR- 24-3p | 22.5501 | 22.61428 | 0.090758 |
| OB 53 | miR- 24-3p | 24.54655 | 25.11835 | 0.808646 |
| OB 53 | miR- 24-3p | 25.69015 | 25.11835 | 0.808646 |
| OB 55 | miR- 24-3p | 24.39612 | 24.48339 | 0.123413 |
| OB 55 | miR- 24-3p | 24.57065 | 24.48339 | 0.123413 |
| OB 57 | miR- 24-3p | 24.32252 | 24.71572 | 0.556073 |
| OB 57 | miR- 24-3p | 25.10893 | 24.71572 | 0.556073 |
| OB 59 | miR- 24-3p | 27.76809 | 27.81625 | 0.068105 |
| OB 59 | miR- 24-3p | 27.86441 | 27.81625 | 0.068105 |
| OB 60 | miR- 24-3p | 24.98585 | 24.9796 | 0.008834 |
| OB 60 | miR- 24-3p | 24.97335 | 24.9796 | 0.008834 |
| OB 63 | miR- 24-3p | 36.91255 | 36.37254 | 0.763679 |
| OB 63 | miR- 24-3p | 35.83254 | 36.37254 | 0.763679 |
| OB 65 | miR- 24-3p | 25.76333 | 25.95754 | 0.274649 |
| OB 65 | miR- 24-3p | 26.15174 | 25.95754 | 0.274649 |
| OB 67 | miR- 24-3p | 22.92936 | 23.27316 | 0.486211 |
| OB 67 | miR- 24-3p | 23.61696 | 23.27316 | 0.486211 |
| OB 69 | miR- 24-3p | 22.96676 | 22.79236 | 0.246646 |
| OB 69 | miR- 24-3p | 22.61795 | 22.79236 | 0.246646 |
| OB 73 | miR- 24-3p | 26.77868 | 26.47215 | 0.433506 |
| OB 73 | miR- 24-3p | 26.16561 | 26.47215 | 0.433506 |
| OB 75 | miR- 24-3p | 24.72328 | 24.69978 | 0.033241 |
| OB 75 | miR- 24-3p | 24.67627 | 24.69978 | 0.033241 |
| OB 77 | miR- 24-3p | 26.1487 | 26.03524 | 0.160455 |
| OB 77 | miR- 24-3p | 25.92178 | 26.03524 | 0.160455 |
| OB 79 | miR- 24-3p | 25.24142 | 25.47975 | 0.337051 |
| OB 79 | miR- 24-3p | 25.71808 | 25.47975 | 0.337051 |
| OB 81 | miR- 24-3p | 24.37283 | 24.68894 | 0.447047 |
| OB 81 | miR- 24-3p | 25.00505 | 24.68894 | 0.447047 |
| OB 83 | miR- 24-3p | 24.66404 | 25.00511 | 0.482341 |
| OB 83 | miR- 24-3p | 25.34617 | 25.00511 | 0.482341 |
| OB 91 | miR- 24-3p | 25.60763 | 25.56927 | 0.054235 |
| OB 91 | miR- 24-3p | 25.53093 | 25.56927 | 0.054235 |
| OB 97 | miR- 24-3p | 25.53602 | 25.49261 | 0.061391 |
| OB 97 | miR- 24-3p | 25.4492 | 25.49261 | 0.061391 |
| OB 99 | miR- 24-3p | 25.75888 | 25.75152 | 0.010403 |
| OB 99 | miR- 24-3p | 25.74417 | 25.75152 | 0.010403 |
| OB 101 | miR- 24-3p | 26.71435 | 26.85592 | 0.20022 |
| OB 101 | miR- 24-3p | 26.9975 | 26.85592 | 0.20022 |
| OB 105 | miR- 24-3p | 23.96197 | 24.00246 | 0.057267 |
| OB 105 | miR- 24-3p | 24.04295 | 24.00246 | 0.057267 |
| OB 107 | miR- 24-3p | 25.9968 | 25.96567 | 0.044032 |
| OB 107 | miR- 24-3p | 25.93453 | 25.96567 | 0.044032 |
| OB 109 | miR- 24-3p | 23.85749 | 23.8749 | 0.024618 |
| OB 109 | miR- 24-3p | 23.89231 | 23.8749 | 0.024618 |
| OB 111 | miR- 24-3p | 25.75942 | 25.65921 | 0.141721 |
| OB 111 | miR- 24-3p | 25.55899 | 25.65921 | 0.141721 |
| OB 113 | miR- 24-3p | 26.9152 | 27.13803 | 0.315124 |
| OB 113 | miR- 24-3p | 27.36085 | 27.13803 | 0.315124 |
| OB 115 | miR- 24-3p | 24.91602 | 25.01052 | 0.133652 |
| OB 115 | miR- 24-3p | 25.10503 | 25.01052 | 0.133652 |
| OB 117 | miR- 24-3p | 22.86763 | 22.88042 | 0.018083 |
| OB 117 | miR- 24-3p | 22.89321 | 22.88042 | 0.018083 |
| OB119 | miR- 24-3p | 25.4733 | 25.40137 | 0.101722 |
| OB119 | miR- 24-3p | 25.32944 | 25.40137 | 0.101722 |
| OB 121 | miR- 24-3p | 24.26099 | 24.34582 | 0.119964 |
| OB 121 | miR- 24-3p | 24.43064 | 24.34582 | 0.119964 |
| OB 123 | miR- 24-3p | 24.70343 | 24.93009 | 0.320543 |
| OB 123 | miR- 24-3p | 25.15675 | 24.93009 | 0.320543 |
| OB 125 | miR- 24-3p | 28.90625 | 28.81951 | 0.122678 |
| OB 125 | miR- 24-3p | 28.73276 | 28.81951 | 0.122678 |
| Controls | | | | |
| Sample Name | Target Name | Cт | Cт Mean | Cт SD |
| OBCQ 1 | miR- 24-3p | 25.31036 | 25.29223 | 0.02564 |
| OBCQ 1 | miR- 24-3p | 25.2741 | 25.29223 | 0.02564 |
| OBCQ 2 | miR- 24-3p | 24.99133 | 24.80589 | 0.262255 |
| OBCQ 2 | miR- 24-3p | 24.62044 | 24.80589 | 0.262255 |
| OBCQ 3 | miR- 24-3p | 24.93812 | 24.95511 | 0.02402 |
| OBCQ 3 | miR- 24-3p | 24.97209 | 24.95511 | 0.02402 |
| OBCQ 4 | miR- 24-3p | 26.40772 | 26.65436 | 0.348806 |
| OBCQ 4 | miR- 24-3p | 26.901 | 26.65436 | 0.348806 |
| OBCQ 5 | miR- 24-3p | 24.11498 | 24.06847 | 0.065781 |
| OBCQ 5 | miR- 24-3p | 24.02195 | 24.06847 | 0.065781 |
| OBCQ 6 | miR- 24-3p | 24.6821 | 24.69884 | 0.02367 |
| OBCQ 6 | miR- 24-3p | 24.71558 | 24.69884 | 0.02367 |
| OBCQ 7 | miR- 24-3p | 25.52591 | 25.72279 | 0.278439 |
| OBCQ 7 | miR- 24-3p | 25.91968 | 25.72279 | 0.278439 |
| OBCQ 8 | miR- 24-3p | 25.24726 | 25.11398 | 0.188496 |
| OBCQ 8 | miR- 24-3p | 24.98069 | 25.11398 | 0.188496 |
| OBCQ 9 | miR- 24-3p | 24.2959 | 24.52161 | 0.319213 |
| OBCQ 9 | miR- 24-3p | 24.74733 | 24.52161 | 0.319213 |
| OBCQ10 | miR- 24-3p | 23.62639 | 23.7959 | 0.239727 |
| OBCQ10 | miR- 24-3p | 23.96542 | 23.7959 | 0.239727 |
| OBCQ11 | miR- 24-3p | 25.7449 | 26.34121 | 0.843304 |
| OBCQ11 | miR- 24-3p | 26.93752 | 26.34121 | 0.843304 |
| OBCQ12 | miR- 24-3p | 24.14561 | 24.14553 | 0.000104 |
| OBCQ12 | miR- 24-3p | 24.14546 | 24.14553 | 0.000104 |
| OBCQ13 | miR- 24-3p | 27.88981 | 28.3616 | 0.66721 |
| OBCQ13 | miR- 24-3p | 28.83338 | 28.3616 | 0.66721 |
| OBCQ15 | miR- 24-3p | 26.2452 | 26.3766 | 0.185837 |
| OBCQ15 | miR- 24-3p | 26.50801 | 26.3766 | 0.185837 |
| OBCQ16 | miR- 24-3p | 27.76013 | 27.92127 | 0.227878 |
| OBCQ16 | miR- 24-3p | 28.0824 | 27.92127 | 0.227878 |
| OBCQ17 | miR- 24-3p | 26.05857 | 26.17039 | 0.158124 |
| OBCQ17 | miR- 24-3p | 26.2822 | 26.17039 | 0.158124 |
| OBCQ19 | miR- 24-3p | 26.53076 | 26.4262 | 0.147857 |
| OBCQ19 | miR- 24-3p | 26.32166 | 26.4262 | 0.147857 |
| OBCQ20 | miR- 24-3p | 25.09686 | 25.11229 | 0.021814 |
| OBCQ20 | miR- 24-3p | 25.12771 | 25.11229 | 0.021814 |
| OBCQ22 | miR- 24-3p | 25.59614 | 25.4208 | 0.247964 |
| OBCQ22 | miR- 24-3p | 25.24547 | 25.4208 | 0.247964 |
| OBCQ23 | miR- 24-3p | 25.873 | 26.42277 | 0.777501 |
| OBCQ23 | miR- 24-3p | 26.97255 | 26.42277 | 0.777501 |
| OBCQ24 | miR- 24-3p | 25.99839 | 25.75285 | 0.347243 |
| OBCQ24 | miR- 24-3p | 25.50731 | 25.75285 | 0.347243 |
| OBCQ25 | miR- 24-3p | 26.49225 | 26.4818 | 0.014783 |
| OBCQ25 | miR- 24-3p | 26.47135 | 26.4818 | 0.014783 |
| OBCQ26 | miR- 24-3p | 26.45198 | 26.54167 | 0.126842 |
| OBCQ26 | miR- 24-3p | 26.63136 | 26.54167 | 0.126842 |
| OBCQ27 | miR- 24-3p | 25.35556 | 25.39438 | 0.054889 |
| OBCQ27 | miR- 24-3p | 25.43319 | 25.39438 | 0.054889 |
| OBCQ28 | miR- 24-3p | 24.30274 | 24.37385 | 0.100562 |
| OBCQ28 | miR- 24-3p | 24.44496 | 24.37385 | 0.100562 |

**Supplementary Table 3.** Raw and Bonferroni-adjusted p-values for miRNAs in patients with obesity

| **miRNA** | **OB vs Control** | |
| --- | --- | --- |
|  | **p_raw** | **Significant_global** |
| hsa-miR-128-3p | **0.003** | **Yes** |
| hsa-miR-21-3p | 0.318 | No |
| hsa-let-7a-5p | 0.156 | No |
| hsa-miR-223-3p | **0.001** | **Yes** |

**Legend**

miRNA: gene symbol; p_raw: raw p-value obtained using the Mann–Whitney test; Significant_global (SG): Significant (Yes)/Not significant (No) depending on whether p_Bonferroni (Bf) < α; Bonferroni-adjusted p-value considering all genes together (n = 4; α = 0.05/4 ≈ 0.0125). OB: Patients with obesity.

**Supplementary Table 4.** Effect size analysis using Cliff’s delta (δ) for miRNAs in patients with obesity

| **Gen** | **|δ|** |
| --- | --- |
| hsa-miR-128-3p | 0.42 |
| hsa-miR-223-3p | 0.49 |

**Legend**

Absolute values of Cliff’s δ (|δ|) indicate the magnitude of the effect size. Effect size interpretation is as follows: |δ| = 0 indicates no difference. 0.33 ≤ |δ| ≤ 0.50 indicates a medium effect. and |δ| = 1 represents the maximum possible difference.

**Supplementary Table 5**. Multivariable logistic regression analysis of factors associated with obesity

| **Variable** | **P-value** | **OR (95% CI)** |
| --- | --- | --- |
| Age | 0.01 | 0.92 (0.86-0.98) |
| DL | 0.03 | 78.40 (1.48-4141.55) |
| miR128 | 0.01 | 2.62 (1.26-5.45) |
| miR223 | 0.01 | 0.02 (0.00-0.34) |

**Legend**

Variables initially included in the model were age, sex, diabetes, hypertension. dyslipidemia (DL), hsa-miR-128-3p, and hsa-miR-223-3p. OR > 1 indicates increased odds of obesity.


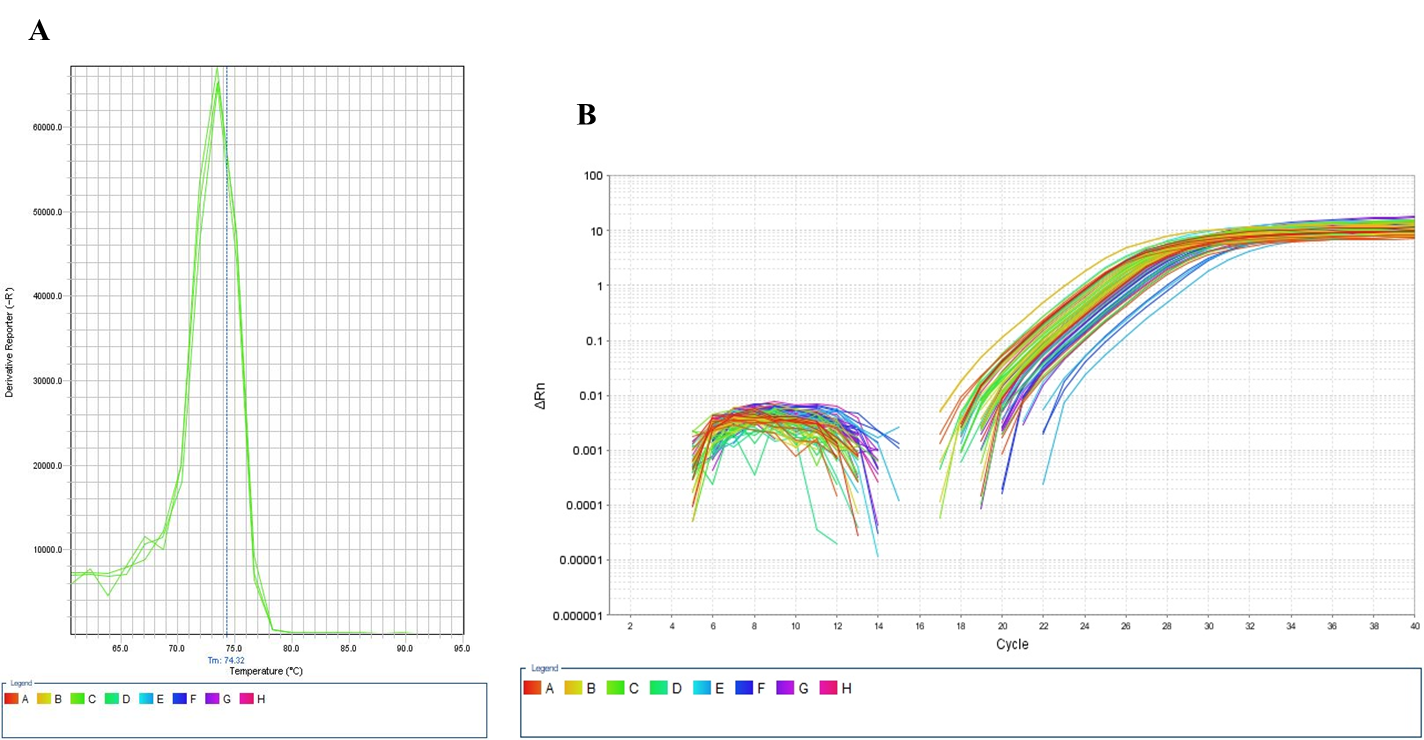


**Supplementary Figure 1.** qPCR analysis of hsa-miR-24-3p in samples from control individuals and patients with obesity. showing (A) the melt curve and (B) the amplification plot.


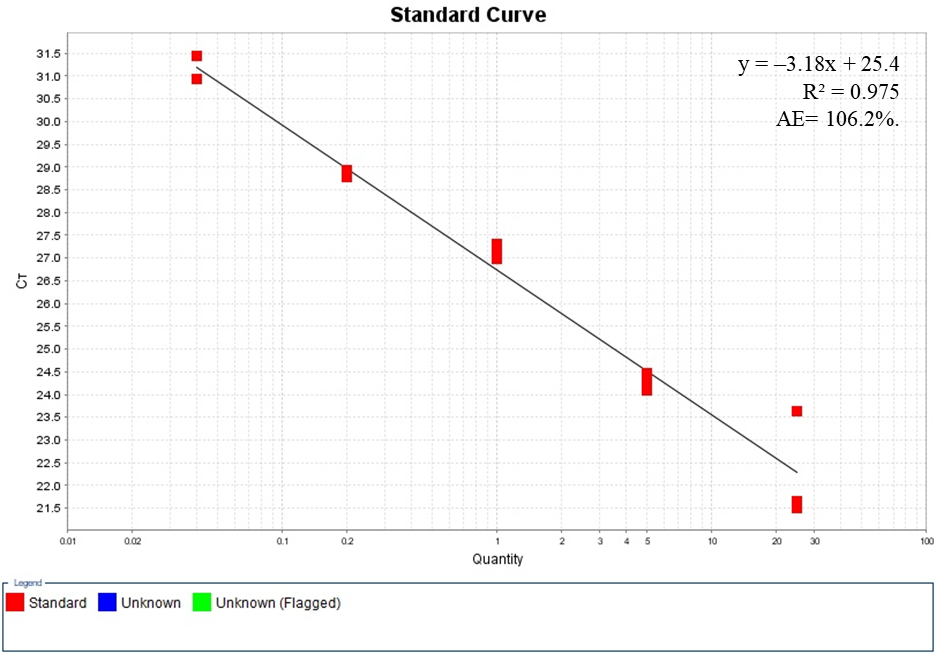


**Supplementary Figure 2. Standard curve for hsa-miR-24-3p amplification.**

The relationship between Ct values and the logarithm of the initial template concentration (serial dilutions) is shown. The regression equation was y = –3.18x + 25.4. with a coefficient of determination (R² = 0.975) and an amplification efficiency of 106.2%.


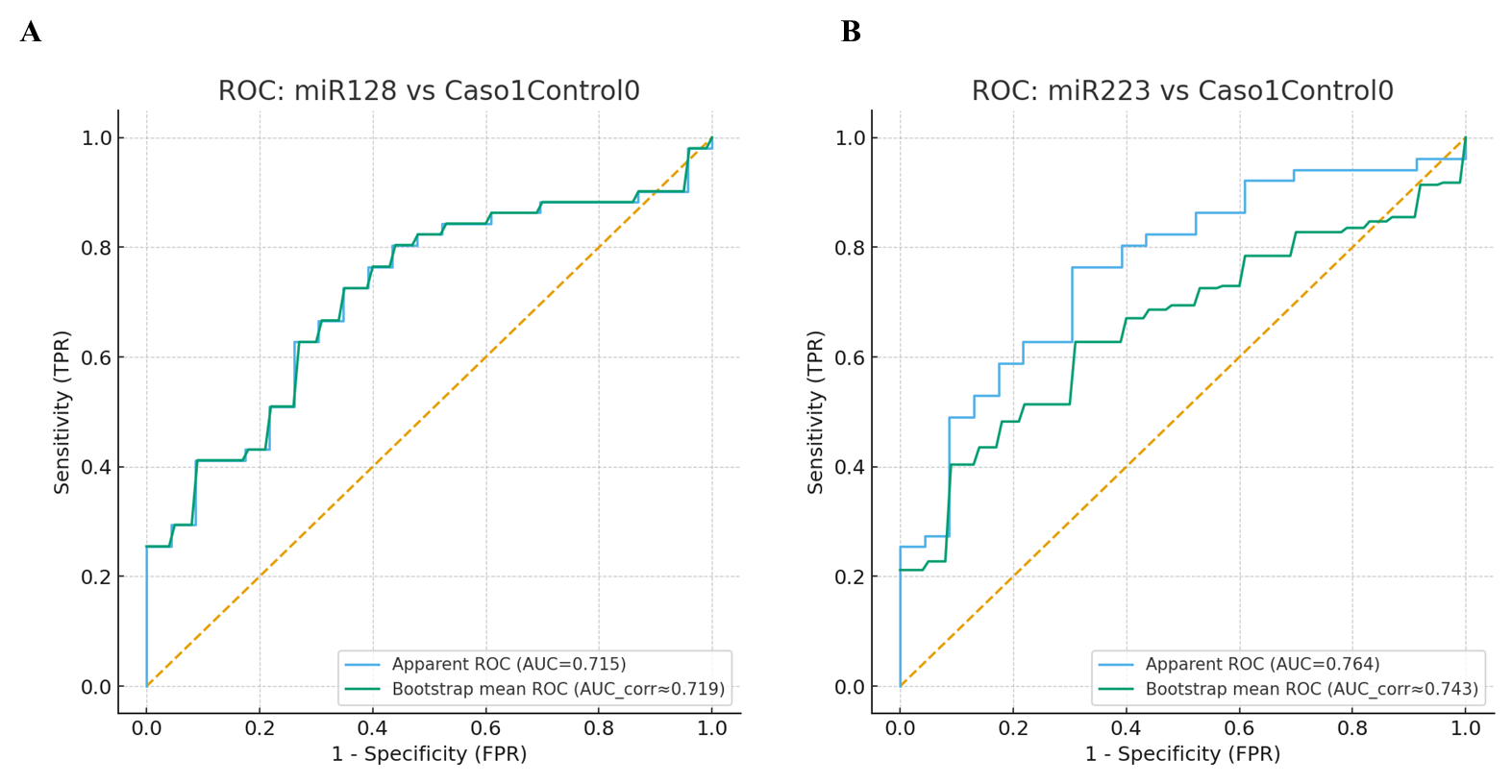


**Supplementary Figure 3: Receiver operating characteristic (ROC) curve for miRNAs hsa-miR-128-3p and hsa-miR-223-3p**. **A.** ROC for hsa-miR-128-3p to discriminate patients with obesity from controls. The apparent ROC is shown together with the bootstrap-validated mean ROC (B=200). Apparent AUC = 0.715; optimism-corrected AUC = 0.719. The diagonal reference line indicates no discrimination. **B.** ROC for hsa-miR-223-3p to discriminate patients with obesity from controls. The apparent ROC is shown together with the bootstrap-validated mean ROC (B=200). Apparent AUC = 0.764; optimism-corrected AUC = 0.743. The diagonal reference line indicates no discrimination. The diagram is a plot of the sensitivity (true-positive rate (TPR) vs. 1-specificity (false-positive rate (FPR)).
